# Application of HPLC-NMR in the Identification of Plocamenone and Isoplocamenone from the Marine Red Alga *Plocamium angustum*

**DOI:** 10.3390/md10092089

**Published:** 2012-09-24

**Authors:** Michael Anthony Timmers, Daniel Anthony Dias, Sylvia Urban

**Affiliations:** School of Applied Sciences, Health Innovations Research Institute (HIRi), RMIT University, GPO Box 2476V, Melbourne, Victoria 3001, Australia; Email: michael.timmers@rmit.edu.au (M.A.T.); ddias@unimelb.edu.au (D.A.D.)

**Keywords:** *Plocamium angustum*, polyhalogenated acyclic monoterpenes, hyphenated spectroscopy, HPLC-NMR, biological activity

## Abstract

A combination of on-line HPLC-NMR and off-line chemical investigations has resulted in the identification of the previously reported polyhalogenated monoterpene plocamenone, together with the new structural analogue isoplocamenone from the crude extract of the marine alga *Plocamium angustum*. On-flow and stop-flow HPLC-NMR analyses (including the acquisition of WET 2D NMR spectra) rapidly assisted in the identification of the major component plocamenone and in the partial identification of its unstable double bond isomer isoplocamenone. Conventional off-line isolation and structural characterization techniques were employed to unequivocally confirm both structures, leading to a structural revision for plocamenone, as well as to obtain sufficient quantities for biological testing.

## 1. Introduction

Crude extracts of marine organisms are frequently composed of a complex mixture of compounds, so any insight into the structure class(es) of the secondary metabolites present is invaluable. To assist in the search and identification of new compounds, hyphenated methodologies (e.g., GC-MS and HPLC-MS) are often employed. The application of HPLC-NMR has been demonstrated as being able to offer valuable insight into the structure class and the range of secondary metabolites present in a crude extract. This has been successfully demonstrated in a number of chemical profiling studies conducted within our research group both on marine and terrestrial extracts and also in monitoring chemical interconversions [1,2,3,4,5]. HPLC-NMR is advantageous in that it has the ability to separate components *in situ* and is a non-destructive technique. This allows for the full recovery of all components for any further studies such as bioassays or mass spectrometry. It is also an invaluable method of analysis for compounds that are unstable [6]. 

**Figure 1:**
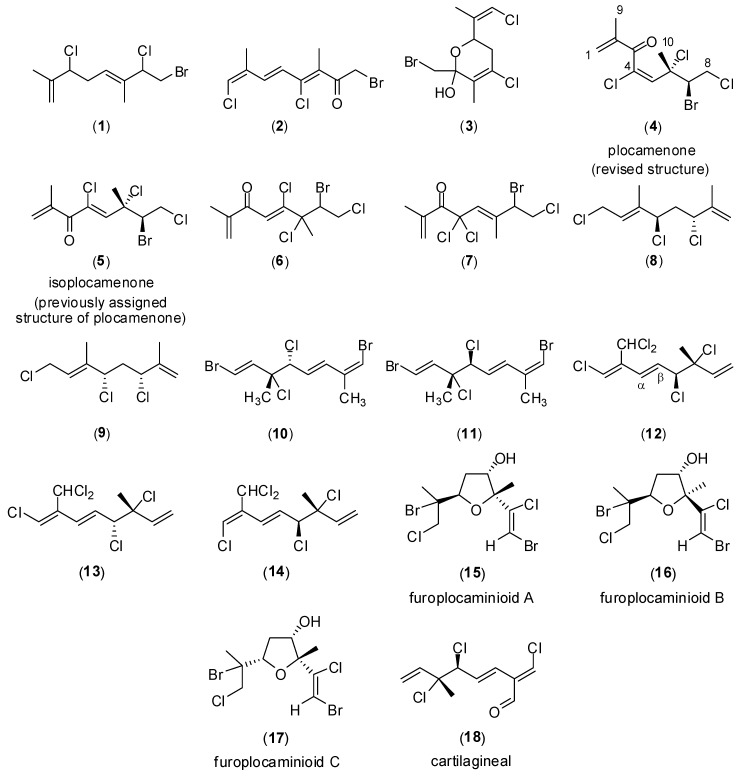
Representative polyhalogenated monoterpenes from marine algae.

A major focus of the Marine And Terrestrial NAtural Product (MATNAP) research group at RMIT University is the study of the chemical diversity and biological activities of Australian marine flora. *Plocamium* is a genus of red algae that is known to produce a vast array of acyclic and cyclic polyhalogenated monoterpenes for which the main differences are associated with the position, orientation and degree of halogen incorporation [1,7]. The species *P. angustum* is endemic to New Zealand and Australia and has not been as widely studied, with only four secondary metabolites having been reported (**1**–**4**) to date (Figure 1) [8,9]. An extract of the red alga *P. angustum* was selected for chemical investigation on the basis of the potent cytotoxicity and significant antimicrobial activities displayed by the crude extract. In addition, chemical investigation of the alga was motivated by the fact that this genus is known to produce a wide array of polyhalogenated monoterpenes, some of which possess bioactive properties, together with the fact that this species has not been extensively studied [10,11,12,13,14,15,16,17].

## 2. Results and Discussion

### 2.1. Secondary Metabolite Profiling of *P. angustum* Using On-Flow HPLC-NMR Analysis

**Figure 2:**
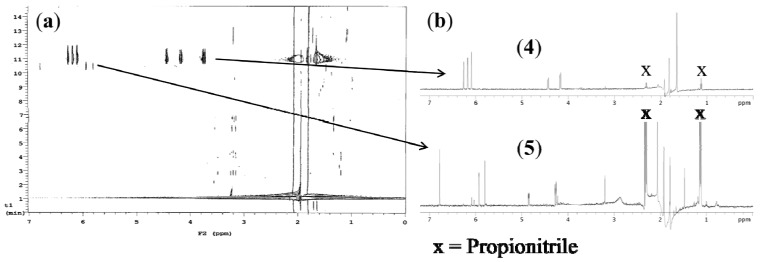
(**a**) On-flow HPLC-NMR contour plot of the methanol fraction of *P. angustum* and (**b**) Stop-flow HPLC-NMR WET 1-D ^1^H NMR spectra of compounds (**4**) and (**5**) acquired using 70% CH_3_CN/D_2_O.

*Plocamium angustum* was extracted as outlined in the *Extraction and Isolation* section. Chemical profiling was conducted on the dichloromethane partitioned fraction employing HPLC-NMR in an attempt to identify the class of secondary metabolites present. HPLC-NMR profiling was achieved using two modes of operation (on-flow and stop-flow). On-flow HPLC analysis clearly indicated the presence of two structurally related secondary metabolites as shown in Figure 2. Given that the genus *Plocamium* is known to produce polyhalogenated monoterpenes, it was proposed that the compounds present in the crude extract were most likely of this structure class. This was also supported by the presence of the deshielded ^1^H NMR chemical shift at δ 4.44 which is characteristic of a halogenated methine.

### 2.2. Secondary Metabolite Profiling of *P. angustum* Using Stop-Flow HPLC-NMR Analysis

Stop-flow HPLC-NMR analysis was also carried out in an attempt to elucidate the structures of the two polyhalogenated monoterpenes, previously observed in the on-flow HPLC-NMR analysis. In the stop-flow mode, selected chromatographic peaks can be trapped in the HPLC-NMR flow cell for an indefinite period, thereby allowing for extended acquisition times and consequently greatly improved sensitivity and resolution in the resulting WET-1D ^1^H NMR spectra. The major secondary metabolite **4** present in the dichloromethane fraction was now subjected to stop-flow HPLC-NMR analysis, whereby acquisition of a WET-1D ^1^H NMR spectrum (1 min) (Figure 2), together with a WET-2D gCOSY (47 min) and a gHSQCAD (4 h) NMR experiment were carried out. On the basis of the WET-2D gCOSY NMR spectrum obtained (see Supplementary Information), protons at δ 4.44 (H-7), δ 4.18 (H-8b) and δ 3.74 (H-8a) all shared correlations, thereby confirming their connectivity. In addition, one of the exocyclic double bond protons at δ 6.18 (H-1b) showed a correlation to the terminal singlet methyl at δ 1.82 (H-9). This NMR data was consistent with the acyclic polyhalogenated monoterpene structure class that has previously been reported from this red alga genus [14,18,19,20]. 

The WET-2D gHSQCAD NMR experiment (see Supplementary Information) showed the presence of an olefinic methine (δ 6.26 to 134.0 ppm), the exocyclic double bond (δ 6.18 and δ 6.09 to 133.2 ppm), a deshielded methine (δ 4.44 to 63.5 ppm), a deshielded methylene (δ 4.18 to 48.0 ppm) as well as a singlet methyl (δ 1.65 to 27.9 ppm). On the basis of the analysis of this NMR data, in consultation with the MarinLit database, it was proposed that the major component was plocamenone, an acyclic polyhalogenated monoterpene previously isolated from *P. angustum* (Figure 1) [21,22]. For this compound to be unequivocally identified, it would be necessary to obtain further NMR spectroscopic evidence of the compound’s connectivity, along with mass spectrometric data. 

The minor component **5** that was observed in the on-flow HPLC-NMR analysis was also subjected to stop-flow HPLC-NMR analysis. This allowed for an extended WET-1D ^1^H NMR spectrum (20 min) to be acquired (Figure 2). It was evident that some of the signals in the WET-1D ^1^H NMR spectrum were either partially or completely suppressed (under the suppressed HDO peak). 

Also the low abundance of **5** meant that any further WET-2D NMR experiments that were acquired in the stop-flow HPLC-NMR mode failed to provide any conclusive connectivity. It was necessary to undertake an off-line isolation and analysis of this compound in order for its complete structure to be elucidated. In addition, the significant cytotoxicity and antimicrobial activities observed for the crude extract of this marine alga further supported the need for an off-line investigation in order to evaluate and determine the nature of the bioactive compound(s).

### 2.3. Off-Line Isolation and Purification of Secondary Metabolites from *P. angustum*

The red alga *P. angustum* was extracted as outlined in the *Extraction and Isolation* section. The dichloromethane partitioned fraction was further fractionated by flash silica chromatography to afford 16 fractions, one of which consisted of the two secondary metabolites previously observed in the HPLC-NMR analyses. This fraction was further purified using semi-preparative reversed phased HPLC to afford plocamenone (**4**) and the minor structurally related secondary metabolite, isoplocamenone (**5**). In the purification of **5**, the compound was isolated either as a separate unstable compound or as a mixture with **4**, since the stability of **5** could be prolonged when isolated as a mixture.

**Table 1:** ^1^H (500 MHz) and ^13^C (125 MHz) NMR data for plocamenone (**4**) in CDCl_3_ and isoplocamenone (**5**) in CD_3_OD.

	Plocamenone (4)				Isoplocamenone (5)			
Position	δ_H_ (*J* in Hz)	δ_C_^a^, mult	gCOSY	gHMBC	δ_H_ (*J* in Hz)	δ_C_^b^, mult	gCOSY	gHMBC
1a	6.13, s	131.2, CH_2_	9	2, 3, 9	5.91, s	129.0, CH_2_	9	2, 3, 9
1b					6.00, s			
2	-	141.7, C	-	-	-	143.5, C	-	-
3	-	191.5, C	-	-	-	192.3, C	-	-
4	-	129.9, C	-		-	133.7, C	-	
5	6.20, s	133.3, CH	-	3, 4, 6, 7, 10	6.84, s	140.8, CH	-	3, 4, 6, 7, 10
6	-	69.0, C	-	-	-	69.7, C	-	-
7	4.36, dd, (3, 9)	62.1, CH	8a, 8b	5, 6, 8, 10	4.97, dd, (2.5, 9)	61.5, CH	8a, 8b	6, 8, 10
8a	3.72, dd, (9, 12)	46.3, CH_2_	7, 8b	7	3.93, dd, (9, 13)	47.1, CH_2_	7, 8b	7
8b	4.24, dd, (3, 12)		7, 8a	6	4.38, dd, (2.5, 13)		7, 8a	6
9	1.94, s	16.5, CH_3_	1	1, 2, 3	1.97, s	18.0, CH_3_	1b	1, 2, 3
10	1.75, s	26.8, CH_3_	-	5, 6, 7	2.05, s	26.2, CH_3_	-	5, 6, 7

^a^ Carbon assignments based on DEPT NMR experiments; ^b^ Carbon assignments based on gHSQCAD and gHMBC NMR experiments.

The structure of plocamenone (**4**) was established by 1D and 2D NMR analysis (Table 1), along with mass spectrometry. 2D NMR experiments confirmed the structure of plocamenone, a marine secondary metabolite previously reported as possessing structure (**5**), isolated from *P. angustum* [8,9,21,22,23].

The structure of plocamenone has been the focus of several structural revisions. Plocamenone was first reported in 1979 and proposed as **6** [8], but in the same year the structure was revised to **5 **(Figure 1) [21]. In 1983 the structure was revised back to **6**, and then in 1984 further revisions to **7** were concluded (Figure 1) [9,23]. This structure was deemed to be incorrect and was again revised back to **5** [22]. In 1997 the structure was again incorrectly described as **7** [24] and finally in 2010 plocamenone was referred to as plocamenone G (**5**) [25]. While all NMR chemical shifts were consistent with those given in literature sources [8,9,23], no complete 2D NMR analysis was reported in the structural elucidation/revisions. We report here the first complete 2D NMR assignment of plocamenone, and the first assignment of its double bond geometry which, has revised the structure of plocamenone from (**5**) to (**4**).

The assignment of *R* or *S* configurations to stereogenic centers in halogenated monoterperenes is not a trivial matter. Fortunately, in 1975 Mynderse and Faulkner observed, for compounds whose structures were confirmed by X-ray crystallography, that the ^1^H NMR chemicals shifts of the position 10 methyl varied in a predictable manner depending on the relative configurations at positions 6 and 7 [7,16,20,26,27,28,29,30]. Crews expanded these rules by making use of the more reliable and more discernable differences in ^13^C NMR chemical shifts for the position 10 methyl carbon atom [28]. Here chemical shift difference of 3 ppm between the (*R. S*) and (*R. R*) configurations have been reported, In particular chemical shifts of ca. δ 28 and 25 are characteristic of 6R*, 7R* (or 6S*, 7S*) and 6S*, 7R* (or 6R*, 7S*) relative configurations, respectively [28]. As such the relative configuration for plocamenone was assigned in 1985 by applying the empirical rules of Mynderse and Faulkner and Crews to the proton and carbon chemical shifts of the methyl group (H 10, δ 1.75; C 10, 26.8 ppm). In particular, the chemical shift of δ 26.8 ppm was consistent with a 6R*, 7R* (or 6S*, 7S*) relative configuration. While these chemical shifts supported an *RS* configuration, attempts to employ calculated ^1^H NMR chemical shifts to resolve the double bond geometry of plocamenone were not fruitful [22]. 

The minor analogue isoplocamenone (**5**) was subjected to similar analyses. However, owing to the instability of this compound, particularly in CDCl_3_, a complete characterization in this NMR solvent was only possible when acquired on mixtures of **5** and **4**. Subsequent re-isolations of **5** were carried out, in an attempt to characterize and identify the compound. However, the compound’s instability continually resulted in rapid degradation being observed after each isolation attempt. ESI-MS analysis of **5** was only possible if undertaken immediately after semi-preparative reversed phased HPLC purification, providing that the fraction containing the compound was not evaporated to dryness. The positive mode ESI-MS of **5** displayed a *m/z* of 335 [M + H]^+^, which was isobaric with that observed for plocamenone (**4**), with an isotopic ratio and splitting also comparable to that of plocamenone (**4**). HR-GC-MS analysis of **5** was carried out on a sample also containing **4** with the chromatographic peak of **5** displaying a *m/z* of 298.9397 [M − Cl]^+^, consistent with C_10_H_12_O^35^Cl_2_^81^Br (calculated for 299.0111). It was noted that this compound’s stability could be prolonged when isolated in the presence of plocamenone (**4**) and so it was decided that this minor secondary metabolite would be isolated as a mixture with **4** to permit further characterization and identification to be carried out. It must be noted that even in a mixture, this minor compound degraded within two weeks. On the basis of the NMR analyses conducted on the mixture in CD_3_OD, complete characterization was possible. Extensive analysis of the 1D and 2D NMR data (Table 1), in combination with the mass spectrometric evidence, quickly established that the minor analogue had the same connectivity as that of plocamenone (**4**) and was an isomer of this compound. 

Owing to the instability of **5**, any further separation and characterization could not be conducted as a means to ascertain the structure of this minor structural analogue. Comparison of the NMR data indicated that the most significant chemical shift differences for plocamenone (**4**) and the minor analogue **5** in CD_3_OD (See Table 2) were observed for the ^1^H NMR chemical shifts at positions 1a, 1b, 5, 7 and 10, whilst the greatest differences in the ^13^C NMR chemical shifts were evident at positions 1, 4 and 5.

**Table 2:** ^1^H and ^13^C NMR chemical shift differences for plocamenone (**4**) and isoplocamenone (**5**) (500 MHz, CD_3_OD).

	plocamenone (4)		isoplocamenone (5)		Differences (ppm)	
Position	δ_H_	δ_C_^a^	δ_H_	δ_C_^a^	∆δ_H_	∆δ_C_
1a	6.17, s	132.3, (t)	5.91, s	129.0, (t)	+0.26	+3.3
1b	6.23, s		6.00, s		+0.23	
2	-	143.3, (s)	-	143.5, (s)		−0.2
3	-	193.2, (s)	-	192.3, (s)		+0.9
4	-	130.5, (s)	-	133.7, (s)		−3.2
5	6.39, s	134.7, (d)	6.84, s	140.8, (d)	−0.45	−6.1
6	-	70.7, (s)	-	69.7, (s)		+1.0
7	4.57, dd, (3, 9.5)	63.5, (d)	4.97, dd, (2.5, 9)	61.5, (d)	−0.40	+2.0
8a	3.80, dd, (9.5, 12)	47.6, (t)	3.93, dd, (9, 13)	47.1, (t)	−0.13	+0.5
8b	4.27, dd, (3, 12)		4.37, dd (2.5, 13)		−0.10	
9	1.93	16.6, (q)	1.97, s	18.0, (q)	−0.04	−1.4
10	1.76	27.6, (q)	2.05, s	26.2, (q)	−0.29	+1.4

^a^ Carbon assignments based on gHSQCAD and gHMBC NMR experiments.

Inspection of ^1^H and ^13^C NMR chemical shifts reported for structurally related halogenated terpenes such as compounds (**8**), (**9**), (**10**) and (**11**) (Figure 1) established that a configurational change of the stereogenic centers only results in small chemical shift differences being observed [7,18,28]. Given that the ^1^H and ^13^C NMR chemical shift differences between plocamenone (**4**) and the minor analogue **5** (Table 2) were much greater than for those observed for changes around stereogenic centers, it was concluded that the structures of **4** and **5** did not differ by a relative configuration change. This ultimately led to the conclusion that the two structures differed in terms of their double bond geometry. Since the double bond geometry of plocamenone had not been previously established, the isolation of this isomer provided an opportunity to resolve this structural feature.

A change in the double bond geometry from *E* to *Z* can have a significant effect on ^1^H NMR chemical shifts. This was apparent when comparing the NMR data for the polyhalogenated compounds (**12**–**14**) (Figure 1) [7]. In trisubstituted olefins it has been documented that the olefinic carbons of the *E* isomer reside at a higher field [31] and in the case of 1,2-disubstituted ethylenes the rule δ*E* < δ*Z* is observed in the ^13^C NMR data of the olefinic carbons [32,33]. In addition, the regiochemistry of furoplocamioids A to C (**15**–**17**) (Figure 1) which possess a 1,2-dihalovinyl system were determined on the basis of the ^13^C NMR chemical shits of the substituted vinyl carbon [34]. A range of model compounds clearly established that the chemical shift of the olefinic proton and associated carbon resonance in trisubstituted olefinic systems are deshielded for a *Z* geometry. This trend is in line with the differences observed in the ^1^H and ^13^C NMR chemical shifts of plocamenone (**4**) and isoplocameone (**5**), for the olefinic H-5 and the associated olefinic carbon resonances at C4 and C5 (Table 2). Further to this, the geometry of the double bond could be assigned by comparison of the observed chemical shifts for the olefinic H-5 to those calculated either through NMR prediction software such as Advanced Chemistry Development (ACD) labs™ and ChemDraw™ [35,36] or those calculated from tables of substituent shielding constants which support compound (**5**) as being the *Z* isomer of plocamenone (**4**) [37,38,39,40]. This trend for both the ^1^H and ^13^C NMR chemical shifts for the *E* isomer to be more shielded than for the corresponding *Z* isomer is also evident in the structure of cartilagineal (**18**) (Figure 1) [37]. 

On the basis of the significant chemical shift differences observed for the olefinic ^1^H and ^13^C NMR resonances for plocamenone (**4**) and the minor analogue as seen in Table 2, it was concluded that the minor analogue **5** was the *Z* double bond geometric isomer of plocamenone (**4**). This meant that the double bond geometry that had been previously depicted in the structure of plocamenone should now be revised to an *.* configuration. This isomer **5** has been attributed the name isoplocamenone and on biosynthetic grounds the same relative configuration is assigned to both isoplocamenone (**5**) and plocamenone (**4**). The ^1^H and ^13^C NMR chemical shifts of the position 10 methyl support an *RS* configuration for both compounds [7].

It is worth noting that HPLC-NMR analysis of *P. angustum* was essential in recognizing the existence of the closely related plocamenone structural analogue **5** and greatly assisted in the eventual isolation and identification of this unstable compound. It may be speculated that isoplocamenone (**5**) could potentially arise as an artefact formed under the extraction conditions. To study this phenomenon, a portion of the frozen alga was extracted with dichloromethane and immediately analysed by analytical HPLC. This confirmed the presence of both plocamenone (**4**) and isoplocamenone (**5**) in the crude extract. This was also supported by the detection of both compounds in the crude extracts analysed by HPLC-NMR which were also conducted on the frozen alga and immediately analysed. On the basis of this evidence, we suggest that both compounds are natural products. The isolation of double bond isomers from the one organism has been documented on other occasions especially in marine algae [7,41,42]. 

Plocamenone (**4**) has been previously reported to exhibit cytotoxicity in the AMES test, as well antibacterial activity towards *Eschericha col.* [21,24]. Plocamenone (**4**) as well as a mixture of plocamenone (**4**) and isoplocamenone (**5**) were subjected to biological screening as detailed in the *Biological Evaluation and Details of Assay.* section. It was observed that the initial plocamenone (**4**) isolated possessed significant cytotoxicity in the P388 murine leukemia cell assay, with an observed IC_50_ of 157.5 ng/mL (Table 3). The IC_50_ for the antitumor compound adriamycin, also known as doxorubicin was 31 ng/mL in this assay. Plocamenone (**4**) also showed activity when tested as an inhibitor of bacterial and fungal pathogens. It was observed that at a concentration of 30 μg/mL, plocamenone (**4**) inhibited the growth of *Bacillus subtilis. Candida albicans* and *Cladosporium resinae* with inhibition zones (radius of inhibition outside the 6 mm diameter application disc) of 10, 2 and 6 mm zones respectively. At a concentration of 30 μg/mL the standard compound chloramphenicol had an inhibition zone of 12 mm against *B. subtilis* and the standard compound nystatin had inhibition zones of 12 mm against both *C. albicans* and *C. resinae*. The biological activity of plocamenone (**4**), as reported in the literature towards *Eschericha coli*, was not observed in this study. It is suggested that this difference is most likely due to a different strain of bacteria being used in the two assays. 

Subsequent biological testing of a highly purified sample of plocamenone (**4**) isolated from the two re-collected specimens of the alga, along with a mixture of plocamenone (**4**) and isoplocamenone (**5**) (present in a ratio of approximately 4:1) both displayed an IC_50_ of <97.5 ng/mL (Table 3). Owing to the instability of isoplocamenone (**5**), this compound could only be tested in a mixture with plocamenone (**4**). However, given the greater abundance of **4** (4:1), the activity observed for the mixture of the two compounds is presumed to be due to the major compound, plocamenone (**4**). 

**Table 3:** Summary of the P388 cytotoxicity of plocamenone (**4**) and isoplocamenone (**5**) isolated from three separate specimens of *P. angustum*.

Specimen Voucher Code	P388 cytotoxiciy IC_50_ (ng/mL)
2006-04 (crude extract)	<4,875
2009-14 (crude extract)	<4,875
2009-15 (crude extract)	22,743
plocamenone (**4**) (obtained from 2006-04)	157.5 *
plocamenone (**4**) (obtained from 2009-14)	<97.5
plocamenone (**4**) (obtained from 2009-15)	<97.5
mixture of **4** and **5** (obtained from 2009-14)	<97.5

* Note: Purity of isolated plocamenone was lower on this occasion.

## 3. Experimental Section

### 3.1. General Experimental Procedures

^1^H, ^13^C and 2D NMR spectra were acquired on a 500 MHz Varian INOVA NMR spectrometer in CDCl_3_ and CD_3_OD with referencing to solvent signals (δ 7.26 and 77.0 ppm and δ 3.30 and 49.0 ppm, respectively). Electrospray (ESI) mass spectra were obtained as detailed in [43]. GC-MS analysis of the sample mixture was carried out on a Varian Saturn 2200 GC-MS-MS and Varian CP-3800 Gas Chromatograph using a Varian VF-5ms 30 m × 0.25 mm ID DF 0f 0.25 GC column with a constant column flow of 1.0 mL/min with a temperature program (0–1 min 60 °C, 20–30 min 250 °C) used. GC parameters include an injector temperature of 250 °C with injector port set to standard split/splitless mode. The mass spectrometer was set to the electron impact (EI) mode with a trap temperature of 200 °C and a mass scan range between 40 and 450 *m/z*. HRGC-MS analysis was carried out on a Waters GCT Premier GC-Time-of-Flight (TOF) system using a BPX5 30 m × 0.25 mm ID column with a constant column flow of 1.0 mL/min with a temperature program (0–0.5 min 40 °C, 18–24 min 320 °C) used. GC parameters include an injector temperature of 250 °C with injector port set to standard split/splitless mode. The mass spectrometer was set to the electron impact (EI) mode with and a mass scan range between *m/z* 40 and 500. Analytical HPLC was performed as in reference [2], on a Phenomenex Gemini ODS (3) C_18_ 100Å 250 × 4.6 mm (5 µm) column with a flow rate of 1.0 mL/min unless specified. Semi-preparative HPLC analyses were performed on a Varian Prostar 210 (Solvent Delivery Module) equipped with a Varian Prostar 335 PDA detector (monitored at λ_max_ 210 and 2 nm) and STAR LC WS Version 6.0 software, an isocratic solvent system (65% CH_3_CN/H_2_O) and a Phenomenex Prodigy ODS (3) 100Å C_18_ 250 × 10 mm (5 µm) column with a flow rate of 3.5 mL/min.

### 3.2. HPLC-NMR Experimental Procedure

Both on-flow and stop-flow HPLC-NMR analysis was performed using isocratic HPLC conditions (70% CH_3_CN/D_2_O) on a Varian Microsorb-MV C_18_ 150 × 4.6 mm (5 µm) column at 1.0 mL/min with detection at λ_max_ 210 and 254 nm. For both on-flow and stop-flow HPLC-NMR experiments, 50 µL injections (3217 µg) of the dichloromethane partitioned fraction were used. For the HPLC-NMR assignment of plocamenone (**4**) and isoplocamenone (**5**) see the Supplementary Information.

### 3.3. Biological Evaluation and Details of Assays

A 2 g portion of the frozen *P. angustum* specimen (voucher specimen 2006-04) was extracted with 3:1 methanol:dichloromethane (40 mL) and evaluated for antitumor, antiviral and antimicrobial activities at a concentration of 50 mg/mL at the University of Canterbury, Christchurch, New Zealand. This crude extract exhibited potent cytotoxicity (IC_50_ < 4874 ng/mL, see Table 3), broad spectrum antifungal activity against *Candida albicans* (10 mm zone of inhibition), *Trichophyton mentagrophyte.* (10 mm zone of inhibition), and *Cladosporium resinae* (20 mm zone of inhibition) as well as selective antibacterial activity against *Bacillus subtilis* (15 mm zone of inhibition). In addition the crude extract also showed a 100% cytotoxic zone towards *Herpes simplex* type 1 and *Polio* virus type 1. Further collections of *P. angustum* (voucher codes 2009-14 and 2009-15) were also subjected to cytotoxicity screening, with one of the two collections having an IC_50_ of <4875 ng/mL, while the other extract possessed an IC_50_ of 22,743 ng/mL. All cytotoxicity results are given in Table 3.

### 3.4. Alga Collections and Identification

A specimen of *P. angustum* was collected on 22 January 2006 from the Borough of Queenscliff near Pt. Lonsdale, Victoria. Two further specimens of the alga were obtained on 13 December 2009 from the same location as the initial collection. The three separate specimens were identified by Dr. Gerald Kraft. Voucher specimens designated the codes 2006-04, 2009-14 and 2009-15 respectively are deposited at the School of Applied Sciences (Discipline of Applied Chemistry), RMIT University.

### 3.5. Extraction and Isolation

The extraction of the frozen specimen of *P. angustum* (175 g) was carried out using 1 L of 3:1 methanol:dichloromethane. The crude extract was decanted and concentrated under reduced pressure and then sequentially partitioned (titruated) into dichloromethane, methanol and water soluble extracts respectively. The dichloromethane fraction (510 mg) was subjected to flash silica column chromatography (20% stepwise elution from petroleum spirits to dichloromethane to ethyl acetate and finally to methanol), resulting in 16 fractions. On the basis of the analytical HPLC analysis and the ^1^H NMR spectrum, one fraction contained the two compounds previously observed in the HPLC-NMR studies. Purification of plocamenone (**4**) and isoplocamenone (**5**) was achieved using semi-preparative reversed phased HPLC employing the method described in the *General Experimental Procedures*, to afford plocamenone (**4**) (30 mg, 0.15%) and isoplocamenone (**5**) (2 mg, 0.01%). While plocamenone (**4**) was observed to be a very stable secondary metabolite, isoplocamenone (**5**) degraded rapidly. Further to this, it was found that the latter degraded much more rapidly once purified than when it was present as a mixture with plocamenone (**4**). Owing to the degradation observed it was necessary to undertake re-collections of the alga. A separate fraction containing a mixture of plocamenone (**4**) and isoplocamenone (**5**) (4:1) was obtained from a combination of the two re-collected specimens of the alga, using the same isolation procedure as described for the initial isolation (45 mg, 0.38%). This mixture was utilized for subsequent NMR analysis as well as for the biological screening.

### 3.6. Off-Line Characterisation of **4** and **5**

Plocamenone (**4**) [(*Z*)-7-Bromo-4,6,8-trichloro-2,6-dimethylocta-1,4-dien-3-one]; isolated as a stable colorless to light brown oil; [α]_D_ −21.5° (*c*, 4.58 mg/mL in CHCl_3_); UV (methanol) ν_max_ 244 nm (ε = 17000); UV profile extracted from HPLC using photodiode array detection (CH_3_CN/H_2_O) 236 nm; IR (film) λ_max_ 2960, 1670, 1450, 1349, 1092, 947 cm^−1^; ^1^H NMR (500 MHz), ^13^C (125 MHz), gCOSY, gHSQCAD and gHMBC NMR assignments are detailed in CDCl_3_ (see Table 1) and in CD_3_OD (See Table 2); ESIMS (positive, 25 V) *m/z* 335 [M + H]^+^, 299 [M − Cl]^+^, 219 [M – Cl − Br]^+^; GC-MS (EI); *m/z* 299 [M − Cl]^+^, 191 [M − C_2_H_3_BrCl]^+^, 169, 156 [M − C_2_H_3_BrCl_2_]^+^, 127, 91, 77, 69, 51; HR-GC-MS (EI); *m/z* 298.9050 [M − Cl]^+^ (calculated for C_10_H_12_O^35^Cl_2_^81^Br: *m/z *299.0117), 216.9908 [M – Cl − Br]^+^, 190.9774 [M − C_2_H_3_BrCl]^+^, 169.0190, 156.0127 [M − C_2_H_3_BrCl_2_]^+^, 77.0285, 69.0248.

Isoplocamenone (**5**) [(*E*)-7-Bromo-4,6,8-trichloro-2,6-dimethylocta-1,4-dien-3-one]; isolated as an unstable colorless to light brown oil; UV profile extracted from HPLC using photodiode array detection (CH_3_CN/H_2_O) 240 nm; ^1^H NMR (500 MHz), ^13^C (125 MHz), gCOSY, gHSQCAD and gHMBC NMR assignments are detailed in CD_3_OD (See Table 1) and in CDCl_3_ (see Supplementary Information); ESIMS (positive, 25V) *m/z* 335 [M + H]^+^, 299 [M − Cl]^+^, 219 [M − Cl − Br]^+^; GC-MS (EI); *m/z* 299 [M − Cl]^+^, 191 [M − C_2_H_3_BrCl]^+^, 169, 156 [M − C_2_H_3_BrCl_2_]^+^, 127, 91, 77, 69, 51; HR-GC-MS (EI); *m/z* 298.9397 [M − Cl]^+^ (calculated for C_10_H_12_O^35^Cl_2_^81^Br: *m/z* 299.0117), 217.0148 [M − Cl − Br]^+^, 191.0016 [M − C_2_H_3_BrCl]^+^, 169.0419, 156.0326 [M − C_2_H_3_BrCl_2_]^+^, 77.0365, 69.0311.

## 4. Conclusions

HPLC-NMR chemical profiling of the red alga *P. angustum* with subsequent off-line HPLC isolation resulted in the identification of plocamenone (**4**), together with a new, unstable double bond isomer, isoplocamenone (**5**). The complete structures of plocamenone (**4**) and isoplocamenone (**5**) were established on the basis of extensive 2D NMR analysis and by recognizing chemical shift trends for closely related trisubstituted olefins. This represents the first complete structure assignment for plocamenone (**4**) leading to a structural revision for this compound, along with its selective antibacterial activity towards *Bacillus subtilis* and moderate broad spectrum antifungal activity. The use of HPLC-NMR to conduct this study clearly demonstrated that it is an invaluable method for the analysis of unstable compounds. 

## Supplementary Files

Supplementary File 1: PDF-Document (PDF, 318 KB)
