# Supplementary material for: Application of HPLC-NMR in the Identification of Plocamenone and Isoplocamenone from the Marine Red Alga Plocamium angustum
Source: Mar Drugs. 2012 Sep 24;10(9):2089–102. doi: 10.3390/md10092089 (PMC3475275; doi:10.3390/md10092089)
Supplement: Supplementary File 1: — PDF-Document (PDF, 318 KB) [file marinedrugs-10-02089-s001.pdf]

Supplementary Information

Figure S1. Stop-flow HPLC-NMR WET 2-D gCOSY NMR spectrum of plocamenone (**4**).

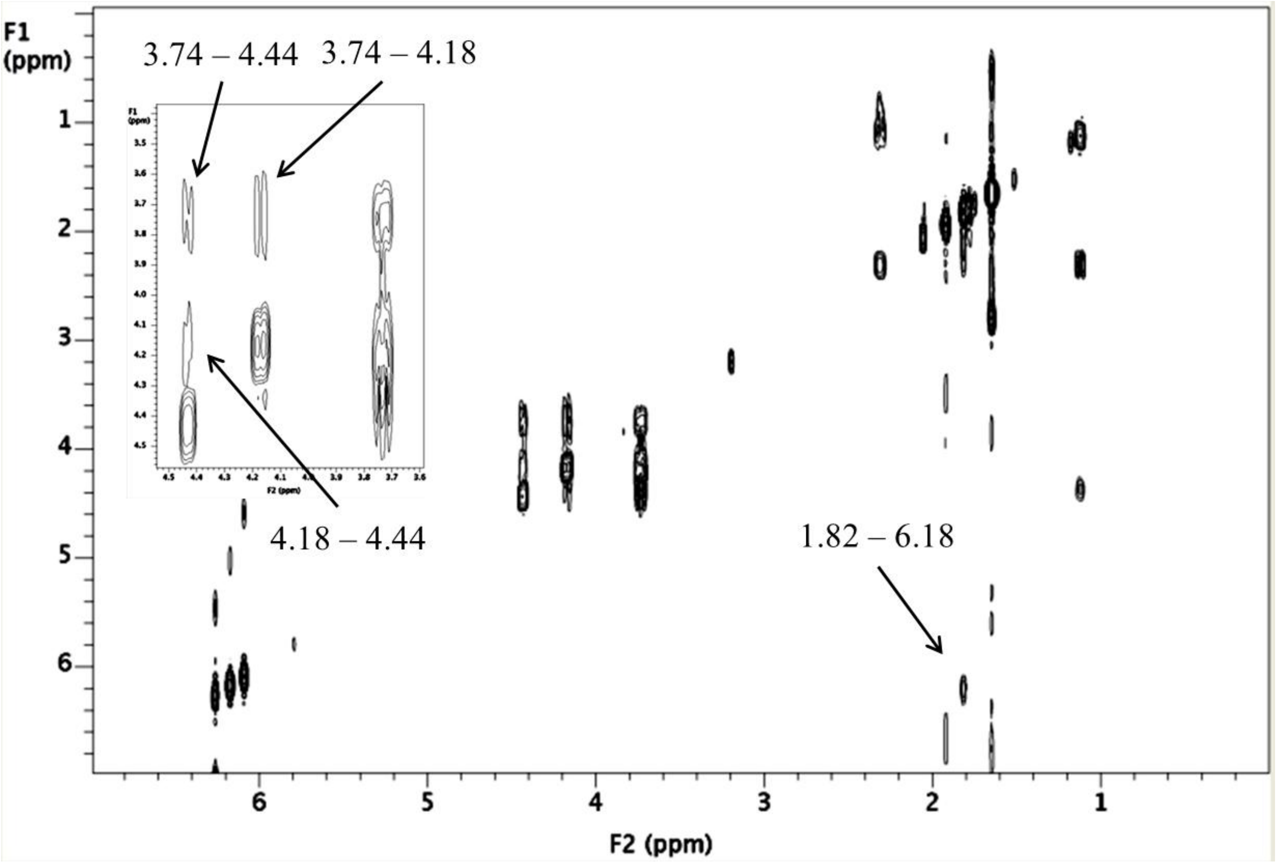

Figure S2. Stop-flow HPLC-NMR WET 2D gHSQCAD NMR spectrum of plocamenone (**4**).

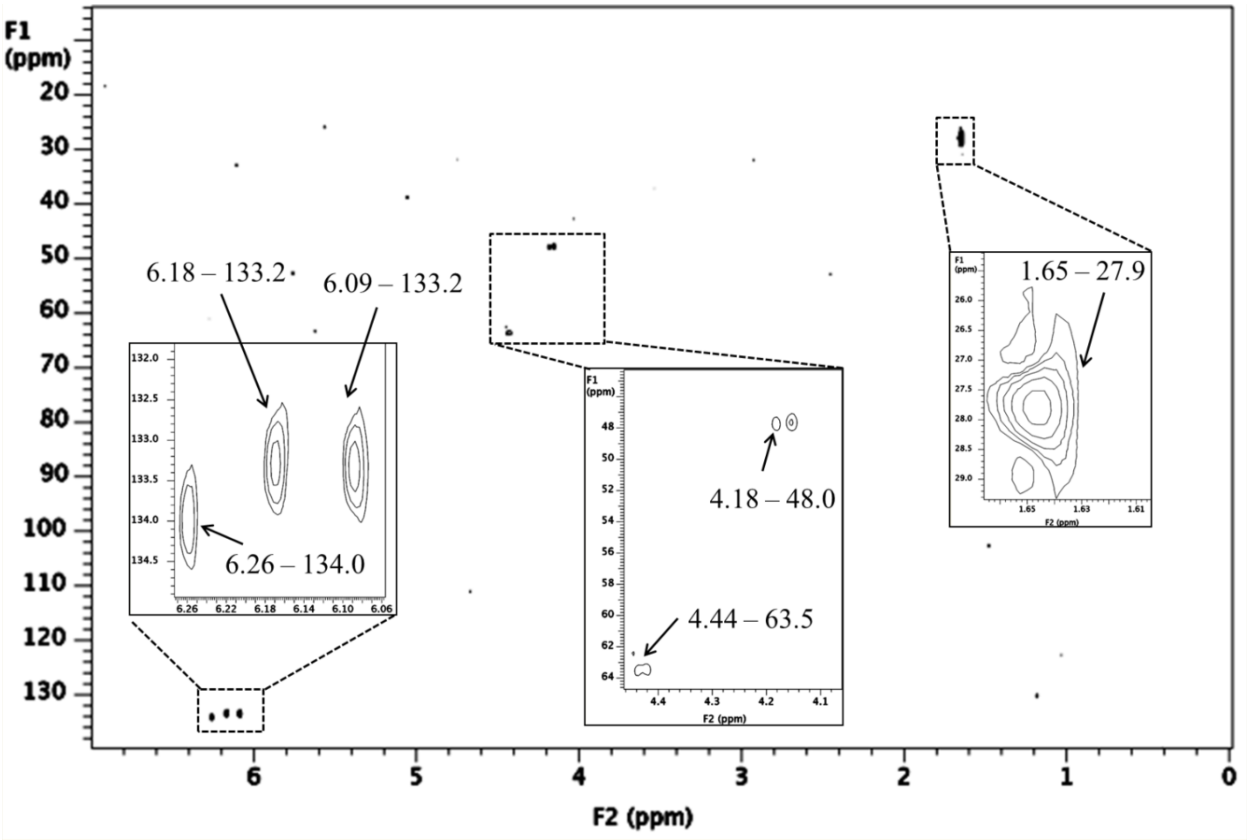

HPLC-NMR Characterization of Plocamenone (**4**) *and Isoplocamenone* (**5**)

HPLC-NMR assignment of plocamenone (**4**) from stop-flow HPLC-NMR: HPLC-NMR (^{1}H, 500 MHz, as detailed in the *Results and Discussion*, δ, ppm); 6.26 (s, H-5), 6.18 (s, H-1b), 6.09 (s, H-1a), 4.44 (dd, *J* = 2.5, 9.5 Hz, H-7), 4.18 (dd, *J* = 2.5, 13 Hz, H-8b), 3.74 (dd, *J* = 9.5, 13 Hz, H-8a), 1.82 (s, H9)* (* indicates signal was partially suppressed), 1.65 (s, H-10); HPLC-NMR (^{13}C, 125 MHz), as detailed in the *Secondary Metabolite Profiling of* P. angustum *Using Stop-Flow HPLC-NMR Analysis* section obtained from the WET-2D HSQC NMR experiment (δ, ppm); 134.0 (d, C5), 133.2 (t, C1), 63.5 (d, C7), 48.0 (t, C8), 27.9 (q, C10), all other carbons not observed.

HPLC-NMR assignment of minor analogue isoplocamenone (**5**) from stop-flow HPLC-NMR: HPLC-NMR (^{1}H, 500 MHz, as detailed in the *Results and Discussion*, δ, ppm): 6.78 (s, H-5), 5.93 (s, H-1b), 5.80 (s, H-1a), 4.85 (dd, *J* = 2.0, 9.5 Hz, H-7), 4.27 (dd, *J* = 2.0, 12.5 Hz, H-8b), 2.06 (s, H10), 1.92 (s, H-9)* (* indicates signal was partially suppressed), H-8a suppressed.

Off-line NMR data of isoplocamenone (**5**) [(*E*)-7-Bromo-4,6,8-trichloro-2,6-dimethylocta-1, 4-dien-3-one]; ^{1}H NMR (500 MHz, $CDCl_3$, δ, ppm): 6.70 (s, H-5), 5.92 (s, H-1b), 5.86 (s, H-1a), 4.85 (dd, *J* = 3.0, 9.0 Hz, H-7), 4.32 (dd, *J* = 3.0, 12.0 Hz, H-8b), 3.82 (dd, *J*=9.0, 12.0 Hz, H-8a), 2.03 (s, H9), 2.00 (s, H-10); ^{13}C NMR (obtained from gHSQCAD and gHMBC NMR experiments, $CDCl_3$, ppm): 192.4 (s, C3) 144.2 (s, C2), 139.5 (d, C5), 133.7 (s, C4), 127.8 (t, C1), 69.8 (s, C6), 59.5 (d, C7), 45.6 (t, C8), 26.2 (q, C10), 18.1 (q, C9).
